# Should Tinnitus Patients with Subclinical Hearing Impairment Be Offered Hearing Aids? A Comparison of Tinnitus Mitigation Following 3 Months Hearing Aid Use in Individuals with and without Clinical Hearing Impairment

**DOI:** 10.3390/jcm12247660

**Published:** 2023-12-13

**Authors:** Sebastian Waechter, Maria Olovsson, Petter Pettersson

**Affiliations:** 1 Department of Clinical Science Lund, Logopedics, Phoniatrics and Audiology, Lund University, 221 00 Lund, Sweden; 2 Region Västra Götaland, Habilitation & Health, Hearing Organization, 541 30 Skövde, Sweden

**Keywords:** tinnitus, hearing aids, amplification, subclinical hearing impairment, hearing loss

## Abstract

There is a consensus among tinnitus experts to not recommend hearing aids for tinnitus patients with subclinical hearing impairment. However, this notion is arbitrary, as no previous study has compared the treatment effect of hearing aids on tinnitus distress in patients with and without clinical hearing impairment. In this article, we investigate whether tinnitus patients with clinical and subclinical hearing impairment differ in terms of tinnitus mitigation after hearing aid fitting. Twenty-seven tinnitus patients with either clinical (*n* = 13) or subclinical (*n* = 14) hearing impairment were fitted with hearing aids. All participants filled out the tinnitus functional index (TFI) before hearing aid fitting and after 3 months of hearing aid use. Clinically meaningful reductions in tinnitus distress (−13 TFI points or more) were seen in both groups, and the difference in tinnitus mitigation between tinnitus patients with clinical (mean TFI reduction = 17.0 points) and subclinical hearing impairment (mean TFI reduction = 16.9 points) was not statistically significant (*p* = 0.991). Group differences on the suspected confounding factors of age, sex, time since tinnitus debut, tinnitus distress (TFI score) at baseline, and treatment adherence were statistically insignificant. In light of this, we argue that clinical hearing impairment is not required to achieve meaningful tinnitus mitigation with hearing aids, and that hearing aids could be recommended for tinnitus patients with subclinical hearing impairment.

## 1. Introduction

Tinnitus, i.e., perceiving a sound that is not caused by an external sound source, is a highly prevalent condition [1] which can have negative impact on the patient’s quality of life [2]. According to a recent meta-analysis by Jarach and colleagues [1] focusing on tinnitus prevalence globally, about 10% of all adults experience chronic tinnitus. The authors of the meta-analysis do, however, mention that obtaining adequate data is being challenged by “the subjective nature of any tinnitus assessment”. As there is currently no established objective measure of tinnitus [3], tinnitus researchers and clinicians rely on subjective measures when estimating the prevalence of tinnitus or tinnitus severity. Researchers are typically using yes-or-no questions when assessing the presence of tinnitus (e.g., “During the last months, have you experienced tinnitus which lasts for more than 5 min?”) and validated questionnaires for assessing the degree of perceived tinnitus distress (e.g., the Tinnitus Functional Index, TFI [4]). The lack of an objective measure of tinnitus limits the precision of the quantitative evaluation of tinnitus interventions, which may, in turn, contribute to difficulties in finding a universal cure for tinnitus [5]. As a consequence, tinnitus patients are often told by healthcare professionals that there is nothing to do for their condition [6]. However, there are several interventions that tend to mitigate patients’ tinnitus; one such intervention is hearing aids. 

The tinnitus-mitigating effect of hearing aids was first reported almost 80 years ago [7]. With the improved precision of hearing aid fittings that came with the transition from analog to digital hearing aids approximately 25 years ago, the efficacy of hearing aid amplification as an intervention for tinnitus seems to have further increased [8]. Specifically, Trotter and Donaldson [8] observed the rates of patients experiencing no tinnitus relief with bilaterally fitted hearing aids dropped from 40% in patients receiving analog hearing aids to only 4% in patients receiving digital hearing aids. Even though there are many reports of positive outcomes for tinnitus patients with hearing aid amplification, high-grade evidence for the intervention is absent [9]. A likely reason for this is that it is obvious for most people wearing a hearing aid whether they are receiving amplification of external sounds or not, which constitutes a methodological obstacle for constructing ecologically valid placebo control conditions for hearing aid amplification. Despite this, the direction of the available evidence favors hearing aids for tinnitus patients [10], with greater treatment response seen if the amplification is objectively verified [11]. In light of the positive reports of hearing aid amplification mitigating tinnitus, major guidelines for clinical tinnitus management recommend hearing aids for tinnitus patients, as long as the tinnitus is accompanied with clinical hearing impairment [9]. 

Another challenge for researchers trying to study hearing aids’ effect on tinnitus is the many possible confounding factors. One such possible confounder is age. Neuroplastic reorganization in response to hearing aid amplification is assumed to be one of the reasons why hearing aid use is associated with tinnitus relief [12], but neuroplasticity decreases with increasing age [13], meaning that older tinnitus patients could hypothetically have lower odds of experiencing tinnitus mitigation with hearing aid amplification. Another hypothesis on why hearing aid amplification could result in milder tinnitus is that the amplified sounds could mask tinnitus and ease communication difficulties, which in turn would decrease tinnitus annoyance [12]. If so, it is plausible that individuals with greater hearing impairment experience greater tinnitus mitigation with hearing aids. Researchers have also suggested that early intervention is of importance for avoiding severe tinnitus distress [14], meaning that the time since tinnitus debut may be a variable of significance. As men and women seem to respond differently on a number of tinnitus treatments [15], possible interaction effects with sex should also be considered. Yet another aspect, which seems particularly closely related to intervention outcome, is treatment adherence, i.e., how much the patients are actually using their hearing aids [16]. 

It is uncommon for tinnitus patients to have no signs of hearing impairment at all [17]. However, there is a substantial portion of tinnitus patients whose hearing thresholds (HT) are perfectly fine, or only mildly affected, at 0.5 to 4 kHz [18], which is the frequency region currently used to define the presence or absence of clinical hearing impairment [19]. Specifically, clinical hearing impairment is defined as a pure-tone average (PTA) of 25 dB or poorer at 0.5, 1, 2, and 4 kHz [19]. This implies that many tinnitus patients having a so-called subclinical hearing impairment (i.e., PTA being better than 25 dB) are currently not recommended hearing aids by clinical guidelines [9], despite not having perfect hearing. These clinical guideline recommendations have been echoed by a British panel of 29 tinnitus experts, who reached a consensus agreement (75% of experts agreeing, 18% disagreeing, 7% neither agreeing or disagreeing) that only tinnitus patients with clinical hearing impairment should be fitted with hearing aids [20]. This notion is widely accepted, but empirical research supporting it is currently limited as no study comparing tinnitus mitigation with hearing aid amplification in patients with and without clinical hearing impairment has been published. While there is reason to believe that the degree of hearing impairment may be critical for tinnitus mitigation with hearing aids (see the section regarding possible confounders above), the hypothesis of hearing aid amplification being an inadequate option for tinnitus patients with subclinical hearing impairment has yet to be verified empirically. 

There are, however, recent examples from the literature where tinnitus patients with PTAs around or below the current cut-off for clinical hearing impairment have been fitted with hearing aids. However, results from such studies are difficult to interpret in relation to the above-mentioned consensus agreement. As these studies have focused on other research questions than the present article, the authors have not presented their results in a manner optimized to determine whether tinnitus patients with and without clinical hearing impairment differ in terms of tinnitus mitigation with hearing aid amplification. Instead, results from tinnitus patients with PTAs just above and below the cut-off for clinical hearing impairment have been grouped together (e.g., the entire sample in the study reported by Suzuki et al. [21], and the control groups in the studies reported by Marcrum et al. [22] and Haab et al. [23]). In addition, the actual results from such studies have been ambiguous. Marcrum et al. [22] used PTA as a covariate in their statistical analysis, to control whether the degree of hearing impairment could have an impact on the intervention effect. The correction for PTA was insignificant, which could be interpreted as the grade of hearing impairment being irrelevant for the outcome. However, Marcrum et al. [22] observed no clinically or statistically meaningful reduction in tinnitus distress pre versus post hearing aid fitting on a group level (although positive outcomes were seen in some individual patients), which may indicate limited benefit of hearing aid amplification for the patient group of interest. The authors do, however, mention that the absence of tinnitus mitigation may also have been due to the adopted hearing aid fitting methodology. Marcrum et al. [22] used the hearing aid manufacturer’s own prescription method instead of a standardized one [24,25]), and did not use probe microphone measurements to verify that the prescribed gain was actually delivered to the participants. Both those choices may limit the benefits typically seen with hearing aids [11,26,27,28]. In contrast, Haab et al. [23] and Suzuki et al. [21] reported significant reduction in tinnitus distress after hearing aid fitting. However, comparing hearing status between the study sample of Suzuki et al. [21] and other studies is challenged by Suzuki and colleagues calculating PTAs using the formula that Japan’s Act on Welfare of Physically Disabled Persons grading system of hearing impairment is based on. Compared to the formula that the World Health Organization (WHO) grading system [12] is based on, i.e., the following:

PTA = ((HT at 0.5 kHz) + (HT at 1 kHz) + (HT at 2 kHz) + (HT at 4 kHz))/4(1)

which is used by most other studies, the Japanese formula omits HT at 4 kHz and counts HT at 1 kHz twice. Taken together, these studies do not provide clear clues as to whether or not hearing aids tend to mitigate tinnitus distress in patients with subclinical hearing impairment. Hence, we cannot know if the current recommendations in clinical guidelines may exclude a substantial portion of tinnitus patients from an intervention that could effectively mitigate their condition. On the other hand, even if hearing aids would mitigate tinnitus distress in patients with subclinical hearing impairment, the recommendation to only fit tinnitus patients with clinical hearing impairments with hearing aids could possibly still be justified should the magnitude of tinnitus mitigation with hearing aids be clearly greater in individuals with clinical compared to subclinical hearing impairment. This information is also difficult to retrieve from the mentioned studies.

To put the current classification of hearing impairment in context, it should be mentioned that researchers have raised concerns about the WHO grading system’s 25 dB PTA limit for normal hearing, as it disregards the many individuals experiencing hearing difficulties despite having PTAs lower than 25 dB [29]. For this reason, it has been argued that the current grading system would benefit from a nuanced revision [30]. Patients experiencing hearing difficulties despite having PTAs < 25 dB are rarely offered hearing aids in a clinical setting, but they do, however, seem to benefit from better speech intelligibility with hearing aid amplification when fitted with hearing aids in a research setting [31]. This indicates that the current classification of clinical hearing impairment could be improved, and that patients currently not recommended hearing aids could benefit from amplification. However, little is known whether patients with tinnitus could also benefit from hearing aid amplification despite having HTs that are currently categorized as normal. 

Tinnitus patients with subclinical hearing impairment do, however, also commonly receive auditory stimulation, but delivered via so-called noise generators (several terms have been used for these types of devices in previous literature, including “noisers”, “sound generators”, and “maskers”) instead of hearing aids. Noise generators typically deliver a constant sound (often white noise) regardless of the patient’s audiometric configuration or what sounds occur around the patient. Hearing aids, on the other hand, amplify the externally occurring sounds based on the patient’s audiometric configuration. The U.S. clinical tinnitus guideline from 2014 mentions white or pink noise stimulation as a possible intervention for tinnitus patients with “normal or near-normal hearing thresholds” [32]. However, the more recent German clinical tinnitus guideline from 2021 [33] recommended against sound generators regardless of the patients’ hearing status, due to poor evidence of efficacy. In line with this recommendation, researchers have warned that prolonged exposure to constant white noise may have detrimental effects on the central auditory system [34]. Furthermore, tinnitus patients often seem to have a hard time tolerating white noise stimulation in the long term [35,36]. Among the ones that keep using noise generators solely in the long term, satisfaction with the device tends to decrease over time, in contrast to what is seen in tinnitus patients using hearing aids [36]. In light of the new recommendations against white noise stimulation for tinnitus patients, the efficacy of hearing aid amplification for tinnitus patients with subclinical hearing impairment deserves to be further investigated.

In an attempt to expand the knowledge on this subject, the present study aims to examine (a) whether a clinically significant reduction in tinnitus distress (a reduction of 13 TFI points or more) is achieved with hearing aids in patients with subclinical hearing impairment (PTA < 25 dB), and (b) whether hearing aids’ tinnitus-mitigating effects differ between tinnitus patients with and without clinical hearing impairment. To control for possible confounders, we aim to explore whether groups differ statistically in terms of age, sex, time since tinnitus debut, and treatment adherence.

## 2. Materials and Methods

### 2.1. Participants

To determine appropriate sample size, a power analysis was conducted using G*Power version 3.1.9.7 (Heinrich Heine Universität, Düsseldorf, Germany [37]). An a priori estimation of the required sample was calculated for a two-tailed independent *t*-test, with α-level set to 0.05, and 1—β error probability set to 0.8. In our opinion, the current recommendation of excluding tinnitus patients with subclinical hearing impairment from hearing aids, an otherwise broadly recommended intervention for tinnitus patients, would be reinforced if very clear differences in intervention outcomes would be present in groups of tinnitus patients with versus without clinical hearing impairment. Therefore, we calculated the required sample size to observe a very large effect size, i.e., Cohen’s d = 1.2 [38]. This power analysis indicated a required sample size of 24 participants split into two equally large groups.

Twenty-nine tinnitus patients with no previous experience of hearing aid use were recruited at Lund University Hospital, Lund, Sweden (*n* = 27), and a public audiology clinic in Skövde (*n* = 2), Sweden. Two of the recruited participants (both recruited at Lund University Hospital, Lund, Sweden) were excluded from the study as they never started wearing their hearing aids, which is why any changes in tinnitus distress were considered to be unrelated to their hearing aids. The remaining 27 participants were divided into groups based on whether their pure-tone average (at 0.5, 1, 2 and 4 kHz) was above 25 dB in at least one ear (clinical hearing impairment group, TCHI, *n* = 13) or below 25 dB in both ears (subclinical hearing impairment group, TSHI, *n* = 14). See Table 1 for descriptive statistics for all included participants. 

**Figure 1:**
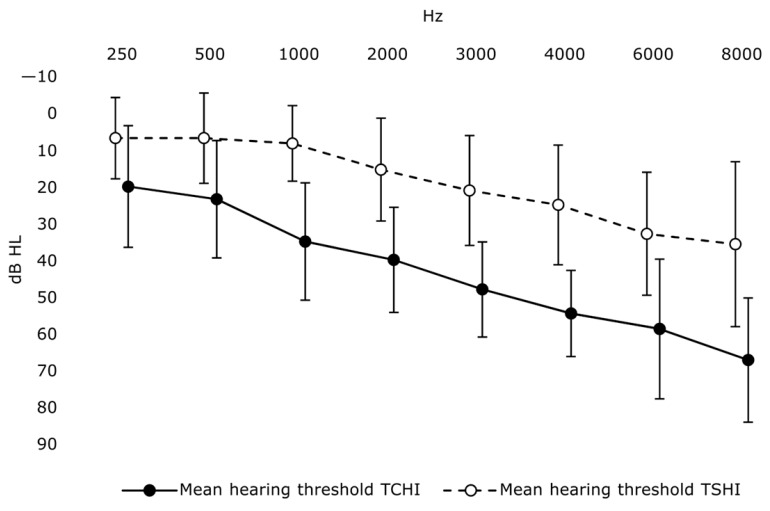
Mean hearing thresholds by group and audiometric test frequency. Error bars display 1 standard deviation. Group symbols are slightly offset from each other at each frequency to facilitate the readability of error bars.

All included participants had experienced chronic tonal tinnitus, with (*n* = 8) or without (*n* = 19) co-occurring tinnitus sounding like noise, for 3 months or longer. Four participants experienced unilateral tinnitus, two participants experienced their tinnitus as a sound inside their head, and the remaining 21 participants experienced bilateral tinnitus. All participants had symmetrical hearing (PTA differed less than 15 dB between best and worst ear), except for two individuals. One of the participants with asymmetrical hearing (30 dB PTA difference between best and worst ear) had PTAs poorer than 25 dB in both ears and was thus included in the TCHI group, while the other participant with asymmetrical hearing (20 dB PTA difference between best and worst ear) had PTAs better than 25 dB in both ears and was thus included in the TSHI group. As some hearing asymmetry was present, with poorer hearing in the tinnitus ear in cases with unilateral tinnitus, we deemed it reasonable to present hearing status as worst ear PTA, which is what PTA hereafter refers to. See Table 1 for descriptive statistics for all included participants. See Figure 1 for group mean HTs at 0.25 to 8 kHz.

### 2.2. Hearing Assessment

Hearing assessments took place in a sound-treated room applying to the international standards for audiometry [39]. HTs were examined by licensed audiologists, conducting standard pure-tone audiometry [39] with calibrated audiometers and headphones.

### 2.3. Hearing Aid Fitting

All participants were bilaterally fitted with hearing aids, except for two participants in the TSHI group with unilateral tinnitus who were unilaterally fitted with one hearing aid for the tinnitus ear only. Hearing aids were fitted in accordance with the prescription method NAL-NL 2 [25]. Since objective verification of the amplification is associated with greater tinnitus mitigation [11], audiologists were recommended to objectively verify the amplification. In 26 cases, audiologists followed this recommendation by adopting so-called real-ear measurements (REM), i.e., probe microphone measurements in the participants’ ear canal to verify that the prescribed gain was achieved. In one participant, the audiologist instead adopted in situ audiometry [40] to verify that adequate amplification was delivered. Participants were recommended to wear the hearing aids for as many of their waking hours as possible.

### 2.4. Evaluation of Tinnitus Distress

The tinnitus functional index (TFI [4]) was chosen as the measure of tinnitus distress at baseline and follow-up. The TFI consists of 25 items, each asking the respondent to indicate to what degree their tinnitus has impacted them during the past week. The 25 items cover eight different themes of tinnitus distress, specifically intrusiveness, sense of control, cognitive difficulties, sleep difficulties, auditory difficulties, relaxation, quality of life, and emotional difficulties. The TFI has been reported to have good validity and test–retest reliability [4]. The choice to use the TFI as an outcome measure was motivated with the intent to make the present results comparable to those of recent studies exploring hearing aids’ tinnitus-mitigating effects [10,11]. As all participants were either native Swedish speakers or fluent in Swedish, a validated Swedish translation of the TFI [41] was used. The validity and test–retest reliability of the validated Swedish translation of the TFI are similar to those of the original TFI [41].

### 2.5. Procedure

First, participants underwent pure-tone audiometry, and filled out the validated Swedish translation of the TFI to indicate tinnitus severity at baseline. TFI responses were recorded via an online survey system (Sunet survey, Artologik, Artisan Global Media, Växjö, Sweden) in all participants but one, who preferred to fill out the questionnaire with pencil and paper. Thereafter, all participants were fitted with commercially available hearing aids manufactured by Signia (Signia/Siemens, Erlangen, Germany), chosen by licensed audiologists based on the participant’s audiometric configuration, personal needs, and preferences. After 3 months of hearing aid use, all participants filled out the validated Swedish translation of the TFI again. In addition, the average daily amount of hearing aid use (hours) was assessed after 3 months of hearing aid use, from the hearing aids’ built-in log. This was assessed in all participants but three, who failed to attend their physical appointment and only filled out the TFI online at follow-up.

### 2.6. Data Analysis

To control for possible confounders, we explored whether the groups differed in terms of age, sex, time since tinnitus debut, baseline tinnitus distress (as measured with the TFI before hearing aid fitting), and treatment adherence (i.e., average daily hearing aid use in hours) during the three months between fitting and follow-up. This was assessed using a MANOVA, with group (TCHI versus TSHI) as independent variable, and age, time since tinnitus debut, and baseline tinnitus distress as dependent variables. Treatment adherence was omitted as a variable in the MANOVA as there were missing data from three participants for this variable. Instead, a separate independent samples *t*-test with group (TCHI versus TSHI) as independent variable and average daily hearing aid use as dependent variable was conducted to control for this potential confounder. Sex was omitted as a variable in the MANOVA as it is a categorical variable. Instead, a separate Chi^2^-test with group (TCHI or TSHI) and sex (female or male) was conducted to control for this potential confounder. To investigate whether there were group differences in terms of tinnitus mitigation (i.e., change in TFI score from baseline to 3 months of hearing aid use), we conducted an independent samples *t*-test with group (TCHI versus TSHI) as independent variable and TFI score change as dependent variable. Statistical analyses were conducted using R version 4.2.2 (R version for statistical analysis, Vienna, Austria, https://www.r-project.org/ (accessed on 1 March 2023)).

## 3. Results

The groups did not differ significantly on any of the tested possible confounders; see Table 2 for sex distribution across groups, as well as group means, standard deviations, and ranges for age, time since tinnitus debut, tinnitus distress at baseline, and treatment adherence, along with *p*-values from statistical between-group comparisons. 

**Figure 2:**
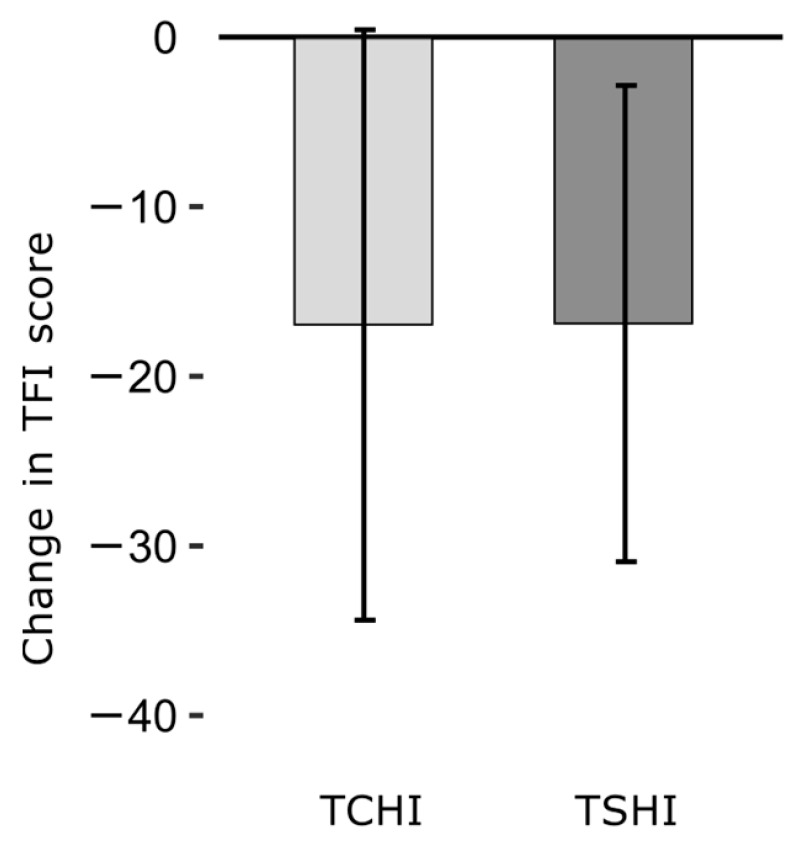
Change in tinnitus distress as measured with tinnitus functional index (TFI) score after 3 months of hearing aid use, in tinnitus patients with (TCHI) and without (TSHI) clinical hearing impairment. Bars represent group mean TFI changes; error bars represent one standard deviation.

TFI changes after 3 months of hearing aid use were very similar in the TCHI (mean TFI change = −17.0, standard deviation = 17.3) and TSHI (mean TFI change = −16.9, standard deviation = 14.0) groups, with no statistically significant difference according to the independent samples *t*-test (t = 0.011, df = 25, *p* = 0.991). See Figure 2 for the visual presentation of the TFI reduction.

## 4. Discussion

Our results indicate that clinical hearing impairment is not required to achieve clinically significant reductions in tinnitus distress (−13 points on the TFI [4]) with hearing aid amplification. Furthermore, we found no statistically significant difference in tinnitus mitigation with hearing aids when comparing patients with clinical (mean TFI change: −17.0) versus subclinical hearing impairment (mean TFI change: −16.9). In light of these findings, we argue that there is evidence suggesting that hearing aids could be considered a treatment option even for tinnitus patients with hearing impairment which is currently classified as subclinical, regardless of tinnitus experts’ opinions [20]. When means are limited, it may, however, still be reasonable to continue prioritizing tinnitus patients with greater hearing impairment for hearing aid fitting, as those patients likely benefit from the intervention to a greater extent in terms of ease of communication where the degree of hearing impairment plays a greater role [42]. In line with this, a tendency of greater reductions on the TFI auditory difficulties subscale (i.e., TFI items relating to the negative impact of tinnitus on the respondents’ perceived ability to hear clearly and follow conversations) was observed in the TCHI compared to the TSHI; see Appendix A, Table A1. However, with evidence indicating positive effects for the patient group of interest, an arbitrary assumption that individuals with subclinical hearing impairment will not experience milder tinnitus with hearing aid amplification should not alone exclude patients from the intervention. 

The statistical between-group comparisons of possible confounders indicated no statistically significant differences in terms of sex distribution, age, time since tinnitus debut, baseline tinnitus distress, or treatment adherence. Despite the absence of statistical significance, there were trends indicating participants with clinical hearing impairment on average were slightly older, had experienced tinnitus for a bit longer, and had a somewhat higher degree of tinnitus distress at baseline compared to participants with subclinical hearing impairment (see Table 1 for means, standard deviations, and ranges on each measure). These trends were, however, expected. Age is a major risk factor for hearing impairment [43], which is why it would be surprising if a non-age-matched groups of patients with and without clinical hearing impairment would not differ a bit in age. Sanchez and colleagues reported that patients with tinnitus and “normal hearing” often develop a hearing impairment after a couple of years [44], which is why it is reasonable that tinnitus patients with clinical hearing impairment typically have experienced tinnitus a bit longer than patients without clinical hearing impairment. As poorer hearing thresholds have previously been reported to be associated with greater tinnitus distress [45,46], the between-group tendency of higher baseline TFI scores among participants with compared to without clinical hearing impairment was expected. We chose not to match groups for these possible confounders, partly as it would have decreased the already limited sample size, and partly because such matching would have decreased the ecological validity of our results as the participants would have represented their population to a lesser extent. An important secondary finding from the present study was that tinnitus patients with and without clinical hearing impairment used their hearing aids to a comparable extent (average daily use was 9.6 and 9.4 h, respectively), indicating that the two groups did not differ in terms of intervention tolerance. 

Admittedly, the present study is limited by its relatively small sample size. However, if a difference in treatment effect should motivate excluding a subpopulation of patients from an otherwise broadly recommended treatment, it could be argued that such differences in treatment effect should be obvious even in a limited sample. Although our power analysis indicated our sample size to be sufficient for detecting very large effect sizes, it should be mentioned that the sample size is insufficient for detecting subtler effect sizes. In addition, only short-term (3 months’ follow-up) effects were studied. Therefore, we cannot rule out the possibility that the effect of the intervention may develop differently longitudinally. In terms of hearing status, the inclusion criteria for the two groups was either having (TCHI) or not having (TSHI) a clinical hearing impairment. Hence, by design, groups’ hearing abilities differed. Specifically, the TCHI had a mean PTA of 37.9 (standard deviation = 10.3), with PTAs ranging from 27.5 to 63.8 within the group, while the TSHI had a mean PTA of 13.5 (standard deviation = 9.1), with PTAs ranging from −2.5 to 23.8 within the group. It should be mentioned that this implies mild to moderate degrees of hearing impairment in most participants in the TCHI. Therefore, we cannot rule out that significant differences in tinnitus mitigation would have been seen, should the group of participants with subclinical hearing impairment and tinnitus (TSHI) be compared to a group of tinnitus patients with more severe hearing impairment. Only including individuals with a mild to moderate degree of hearing impairment in the TCHI was not an active choice, but likely an effect of individuals with severe hearing impairment rarely being first-time hearing aid users nowadays (at least in Sweden, where the present study was conducted). In contrast, being a first-time hearing aid user was an inclusion criterion in the present study, in order to enhance comparability across groups. Given these circumstances, the range of hearing impairment degree in the TCHI was expected. A further limitation of the present study is the absence of a placebo control condition, which is difficult to construct for hearing aids as patients tend to notice if external sounds are amplified or not. From a scientific point of view, this limits the interpretation of the results as the study design does not allow us to investigate whether hearing aid amplification causes tinnitus mitigation. This limitation is highly relevant given tinnitus patients’ susceptibility to placebo effects [47]. It is also possible that tinnitus patients may experience less distress due to their tinnitus just by receiving attention from a trained clinician, or knowing that they are part of a research project. However, tinnitus patients who receive hearing aid amplification in combination with counseling experience greater tinnitus mitigation than those who only receive counseling [48], which makes the hypothetical scenario of the TSHI having achieved comparable tinnitus distress reduction as the TCHI if their reduction had nothing to do with the hearing aid amplification less probable. From a clinical point of view, our results indicate that one of the most commonly provided tinnitus interventions may be suitable not only for patients with a certain degree of hearing impairment. We encourage future research to investigate whether the present findings are to be confirmed in studies with larger sample sizes and longer follow-up durations.

As previous authors have pointed out, there is great variance in tinnitus patients’ response to hearing aid intervention, and efforts should be made to understand the underlying causes for this [10]. In the present study, such variance was seen among individuals both with clinical and with subclinical hearing impairment. In both groups, some experienced no benefit at all, while others’ TFI scores decreased by more than 70% after three months of hearing aid use. Disentangling what parameters seem to affect the individual patient’s response to hearing aid intervention would be of great value, as this would likely come with the opportunity to optimize intervention options. Progress has been made in this field, e.g., [49], but a broader understanding is needed. Even though our results indicate the degree of hearing impairment to be of little predictive value for the magnitude of tinnitus mitigation with hearing aids, we encourage future research to consider possible interaction effects between the degree of hearing impairment and factors assumed to affect the individuals’ responses to hearing aid intervention. The weak statistical power due to the small sample size of the present study limits our abilities to explore differences between the groups in reduction on all specific TFI subscales, i.e., what particular tinnitus-related difficulties were mitigated, in a statistically meaningful way. However, we observed trends where patients with clinical hearing impairment seemed to experience greater reductions on TFI items relating to perceived quality of life and how much their tinnitus interfered with their ability to follow conversations in every-day life, while greater reductions on TFI items relating to the tinnitus intrusiveness and impact on sleep were seen in patients with subclinical hearing impairment (descriptive statistics of TFI subscale reductions are found in Appendix A, Table A1). If these observations are confirmed in a greater sample, they may suggest that individuals with subtle compared to more severe hearing impairment experience tinnitus mitigation with hearing aid amplification due to different reasons. If so, understanding the respective mechanism is key to improving the quality of tinnitus healthcare. In addition, we also encourage future studies to explore to what degree variables such as time since tinnitus debut and treatment adherence are related to tinnitus mitigation, as their relationships with treatment outcome may have implications for what recommendations clinicians should give to tinnitus patients.

### Conclusions

Our results indicate that clinical hearing impairment is not required to achieve clinically significant reductions in tinnitus distress with hearing aid amplification, and that intervention responses are very similar in individuals with and without clinical hearing impairment. Thus, from an intervention response perspective, hearing aids could be offered not only to tinnitus patients with clinical hearing impairment but also to tinnitus patients with subclinical hearing impairment.

## Figures and Tables

**Table 1:** Descriptive statistics for all included participants in total. SD = standard deviation; TFI = tinnitus functional index; PTA = worst ear pure-tone average at 0.5, 1, 2, and 4 kHz.

	Total (*n* = 27)
**Sex**	
Female	10 (37.0%)
Male	17 (63.0%)
**Age (years)**	
Mean (SD)	51.7 (13.1)
Range	32.2–78.6
**Time since tinnitus debut (years)**	
Mean (SD)	14.8 (10.8)
Range	0.3–35.0
**Tinnitus distress at baseline (TFI score)**	
Mean (SD)	46.3 (14.7)
Range	16.4–72.4
**Degree of hearing impairment (PTA)**	
Mean (SD)	25.2 (15.6)
Range	−2.5–63.8

**Table 2:** Between-group differences in possibly confounding variables. SD = standard deviation; TFI = tinnitus functional index.

	TCHI (*n* = 13)	TSHI (*n* = 14)	*p*-Value
**Sex**			0.802 ^1^
Female	4 (30.8%)	6 (42.9%)	
Male	9 (69.2%)	8 (57.1%)	
**Age (years)**			0.111 ^2^
Mean (SD)	55.9 (14.5)	47.8 (10.7)	
Range	35.6–78.6	32.2–61.5	
**Time since tinnitus debut (years)**			0.318 ^2^
Mean (SD)	17.0 (12.0)	12.8 (9.6)	
Range	0.3–35.0	0.3–28.3	
**Tinnitus distress at baseline (TFI score)**			0.101 ^2^
Mean (SD)	51.1 (15.4)	41.8 (13.0)	
Range	23.2–72.4	16.4–64.0	
**Treatment adherence (average daily hearing aid use)**			0.879 ^3^
Mean (SD)	9.6 (4.3)	9.4 (3.7)	
Range	1–14	3–14	

^1^ Calculated with Chi^2^-test; ^2^ calculated with MANOVA; ^3^ calculated with independent samples *t*-test.

## Data Availability

The data are not publicly available due to ethical restrictions. The Swedish Ethical Review Authority has not granted permission to share data, as this was not part of the application.

## Appendix A

**Table A1:** Descriptive statistics of tinnitus functional index (TFI) subscale change after 3 months of hearing aid use for each group. TCHI = tinnitus patients with clinical hearing impairment; TSHI = tinnitus patients with subclinical hearing impairment; TFI-I = TFI intrusiveness subscale; TFI-SoC = TFI sense of control subscale; TFI-C = TFI cognitive subscale; TFI-S = TFI sleep subscale; TFI-A = TFI auditory subscale; TFI-R = TFI relaxation subscale; TFI-QoL = TFI quality of life subscale; TFI-E = TFI emotional subscale.

	TCHI (*n* = 13)	TSHI (*n* = 14)
TFI-I (mean, SD)	−14.4, 18.5	−22.1, 17.1
TFI-SoC (mean, SD)	−12.6, 32.2	−16.2, 22.2
TFI-C (mean, SD)	−13.8, 22.6	−16.0, 14.7
TFI-S (mean, SD)	−7.7, 22.7	−15.0, 27.8
TFI-A (mean, SD)	−26.7, 36.9	−7.1, 25.5
TFI-R (mean, SD)	−26.4, 25.4	−31.7, 26.4
TFI-QoL (mean, SD)	−22.5, 27.1	−12.9, 16.6
TFI-E (mean, SD)	−9.7, 28.2	−15.2, 18.7
